# Thoracic sympathectomy for the treatment of primary axillary hyperhidrosis: systematic review and proportional meta-analysis

**DOI:** 10.1080/07853890.2021.1953126

**Published:** 2021-07-20

**Authors:** Gilmar Felisberto, Antônio José Maria Cataneo, Daniele Cristina Cataneo

**Affiliations:** a Department of Surgery, Post-Graduation Program in Surgery and Translational Medicine, Botucatu School of Medicine, São Paulo State University, UNESP, São Paulo, Brazil; b Department of Surgery, Division of Thoracic Surgery, Botucatu School of Medicine, São Paulo State University, UNESP, São Paulo, Brazil

**Keywords:** Hyperhidrosis, axilla, sympathectomy, video-assisted thoracic surgery, systematic review

## Abstract

**Introduction:**

Primary hyperhidrosis is a disorder that involves excessive sweat production, which has a negative impact on the quality of life.

**Objective:**

To evaluate the effectiveness and safety of video-assisted thoracoscopic sympathectomy (VATS) for treating primary axillary hyperhidrosis (PAH) and determine which level of ganglion resection offers the best outcome.

**Method:**

This was a systematic review and proportional meta-analysis of observational studies. The result was evaluated for satisfaction, control of symptoms, compensatory sweating and complications. A subgroup analysis was performed to compare the sympathetic trunk resection at high and low levels.

**Results:**

Thirteen studies were selected with a total of 1463 patients. The satisfaction rate was 92% (95% CI = 88–95%, *I*^2^=47.5%), the symptom control rate was 96% (95% CI = 93–99%, *I*^2^=48.2%), and the presence of compensatory sweating could not be assessed because of high heterogeneity among studies. The complications were rare.

**Conclusion:**

This review demonstrated that thoracic sympathectomy by VATS is a viable and safe option for the treatment of PAH. There was no difference between high and lower levels of resection. However, the estimation of the effect is quite uncertain because the quality of evidence was extremely low.

**Key message**
- Pure axillary hyperhidrosis has great potential to compromise quality of life.
- Surgery should be indicated only when clinical treatment fails.
- Thoracic sympathectomy by video-assisted thoracoscopy is a viable and safe option for the treatment of primary axillary hyperhidrosis.

## Introduction

Primary hyperhidrosis is the most common form of sweat gland disorder and is not related to body thermal control and is usually triggered by emotional stress. It typically has a focal and bilateral distribution, and the generalized form is less common. It primarily affects the axillae, hands and feet and less often the craniofacial regions [1]. Most individuals have symptoms at more than one site with the palmar–plantar combination being the most frequent [2]. The prevalence of this disorder is variable with values ranging from 0.6% to 17.9% of the general population being reported [3–5]. Both sexes are similarly affected and the onset of symptoms usually occurs in adolescence between the ages of 14 and 25 years [3,4].

Although a benign condition, primary hyperhidrosis has considerable impact on the quality of life of affected individuals because it compromises daily activities, work and social interaction. Moreover, primary hyperhidrosis can be as harmful as diseases such as psoriasis, rheumatoid arthritis, multiple sclerosis and end-stage kidney disease [4,6]. The hyperhidrosis treatment is multifactorial, including several topical, surgical and behavioural methods [7]. Currently, surgical treatment is indicated after the failure of conservative treatment options, and the primary surgical option is thoracic sympathectomy [8,9]. Thoracic sympathectomy yields better results when symptoms are not localized solely in the axillary region [8]. There is no consensus on the best therapeutic method for patients who only have axillary symptoms [7,8].

Thus, the aim of this review was to analyse the effectiveness and safety of thoracic sympathectomy by video-assisted thoracoscopy for treating primary axillary hyperhidrosis (PAH) and to determine the level of sympathetic resection that yields the best results.

## Method

The method used followed the recommendations and guidelines provided by the Cochrane Collaboration and PRISMA Statement for conducting and reporting a systematic review.

### Eligibility criteria

For this review, the initial search for randomized clinical trials yielded only one such study. Thus, prospective and retrospective observational studies that included the following eligibility criteria were accepted.

#### Participants

Adult patients with pure axillary hyperhidrosis.

#### Intervention

Treatment performed through thoracic sympathectomy by videothoracoscopy.

#### Outcomes

Satisfaction, control of symptoms, compensatory sweating and treatment-related complications.

The first three results were evaluated using structured interviews, self-administered questionnaires, telephone calls, letters, graduated visual scales and spontaneous testimonials. The fourth outcome was assessed by analysing medical records.

### Search strategy for identifying studies

The following databases were searched: Medline (1966–2020), Cochrane Register of Controlled Trials (2020), Embase (1980–2020) and Lilacs (1988–2020). Moreover, the gray literature was consulted through the following databases: Clinical Trials, ISI Web of Knowledge and British Library. All references of the included studies were confirmed, and there was no language restriction. For this review, the basis of the search strategy, which was adapted to each database, was formulating the clinical question: Is thoracic sympathectomy performed by video-assisted thoracoscopy a safe and effective option for treating patients with pure axillary hyperhidrosis?

## Selection of studies and data collection

The selection of studies was independently performed by two reviewers (GFJ and AJMC). After excluding duplicate studies, the titles and abstracts were analysed for removing studies irrelevant to this review. All potentially eligible studies were read in full and their inclusion or exclusion was defined with the aid of the standard form. Disagreements were resolved by consensus, and a third reviewer (DCC) was available in case of a divergence.

### Subgroup analysis

Patients who underwent sympathetic trunk resection at high and broad level (at any of these levels: T1, T2 and T3, associated or not with T4) and at low level (T4 and/or T5) were separately analysed.

### Statistical analysis

The outcomes analysed in the meta-analysis were treated as dichotomous variables. For the proportional meta-analysis, which compared the results obtained within only one group of patients, the results were presented as a forest plot, in which each line represents one study included in the analysis. The study’s effect is represented by a square with the size of the square being the weight of the study in the meta-analysis. The estimate of the combined effect is represented by a diamond at the base of the graph. In this analysis, an overlap of the confidence intervals of the different interventions indicates that there was no statistical difference between the groups. However, absence of overlap shows that there was a difference between the evaluated groups. To quantify the inconsistencies among the studies included in the meta-analysis, the heterogeneity test *I*^2^= [(*Q* – df)/*Q*] × 100% was used with *Q* being the chi-square and df the degree of freedom. Whenever heterogeneity was >0, the random effect was used. All analyses were performed using the StatisDirect 3.2.8.

### Evaluation of the quality of the included studies and of the quality of evidence

Evaluation of the quality of the included studies was performed using the Newcastle–Ottawa scale, in which three domains are analysed: selection, comparability and outcomes. In terms of the selection and outcome domains, which have four and three subdomains, respectively, each study can receive only one point per subdomain. In terms of the comparability domain, two points are possible; thus, the maximum score for each study is nine points. The quality of evidence was analysed using the tool proposed by GRADE, available online at https://gdt.gradepro.org/app/.

## Results

**Figure 1.**
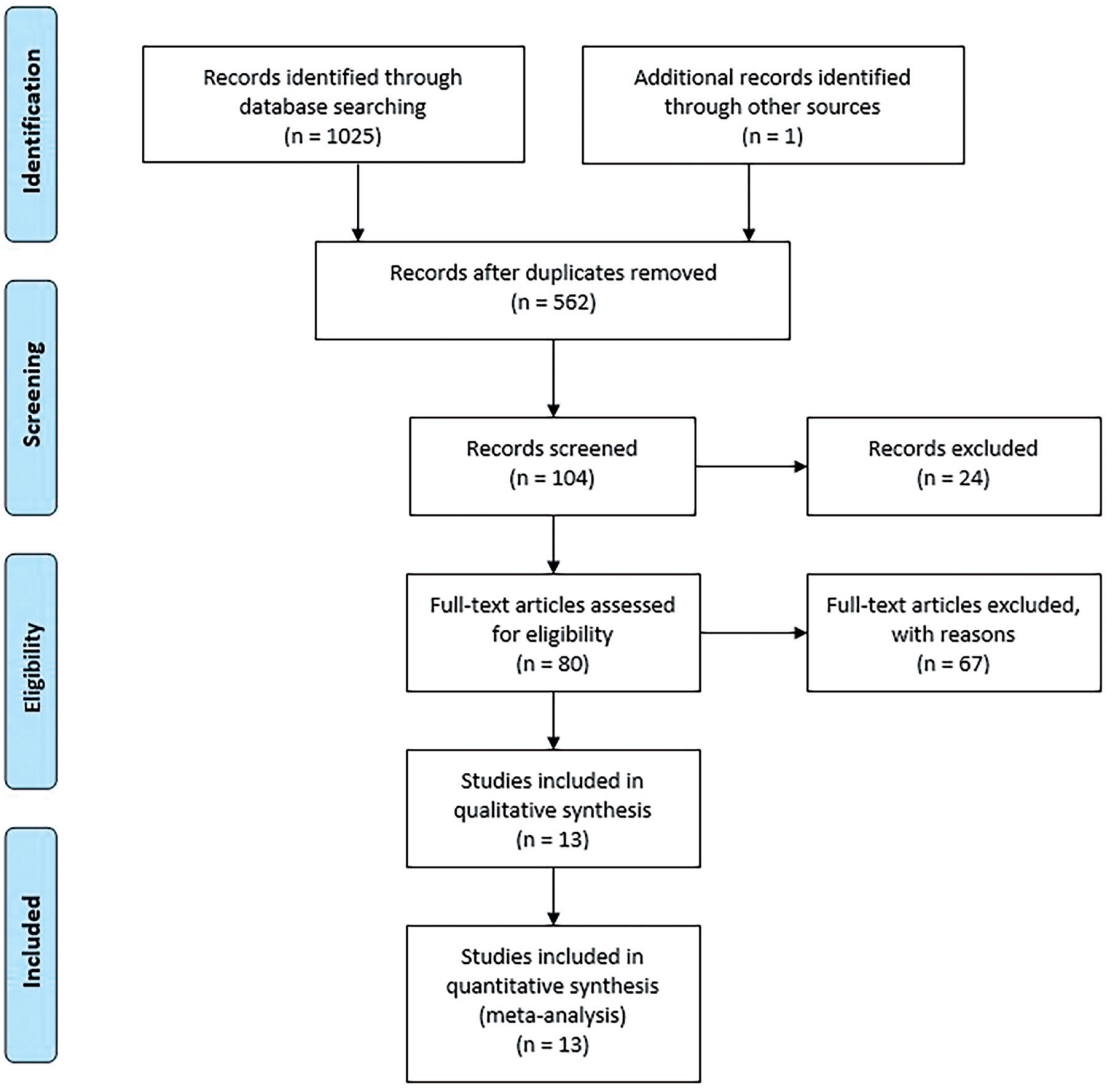
Flowchart of literature search.

The search conducted in December 2019 identified 253 studies in Pubmed, 123 studies in the Embase database, 318 studies in the Bireme database, 39 studies in the Web of Science and 292 studies in the British Library. No articles were found in the Cochrane and Clinical Trials databases. After excluding duplicate publications, there were 562 remaining studies. After analysing titles and abstracts, 80 studies were selected for full analysis, of which 13 studies were selected for this review (Figure 1).

Only one of the 13 selected studies was randomized and prospective [10]. The others were longitudinal studies, and three studies were prospective [11–13] and 10 were retrospective [14–22]. The mean age of the participants was reported in six studies (28 years). The follow-up time widely varied among the studies, with the shortest period being 12 months and the longest 16.1 years. The total number of patients evaluated was 1463. It was possible to determine the sex of 768 individuals (52.49%), 544 (70.83%) were women and 224 (29.17%) were men. Table 1 shows the primary characteristics of the selected studies, and Table 2 shows the results of the evaluation of the quality of the studies according to the Newcastle–Ottawa scale.

**Table 1.** Characteristics of selected studies.

Author	Origin	Period	Number of participants	Sex (M, F)		Follow-up (months)	Sympathectomy level
Boscardim et al. [14]	Brazil	2003/2007	118	26	92	12	T4/T5
Chou [15]	Taiwan	–	28	8	20	35.3	T5
Raposio et al. [20]	Italy	–	9	–	–	15 months	T4
Hsu et al. [16]	Taiwan	1996/2000	171	37	134	22.5	T3/T4_T4_T4/T5
Lin [18]	Taiwan	1997/1998	26	10	16	31.3	T3/T4
Lin et al. [19]	Taiwan	1993/2000	480	–	–	51.7	T3/T4
Munia et al. [10]	Brazil	–	64	14	50	12	T3/T4_T4
Rex et al. [21]	Sweden	1989/1996	93	–	–	26	T2/T4
Ribas Milanez de Campos et al. [22]	Brazil	2000/2005	276	106	170	21.6	T3/T4_T4
Schmidt et al. [11]	Germany	2000/2003	85	23	62	24	T2/T4_T3/T5
Sciuchetti et al. [12]	Italy	2003/2006	51	–	–	13	T3/T4
Leao et al. [17]	Brazil	1999/2003	21	–	–	36	T2/T4
Zacherl et al. [13]	Austria	1965/1996	41	–	–	16.1 (years)	T1/T4

M: male F: female.

**Table 2.** Newcastle–Ottawa scale.

	Selection				Comparability	Outcomes			
Study	Representativeness	Selection of non-exposed group	Investigation	Outcome absent at the beginning	Comparability	Evaluation results	Segment duration	Adequacy of follow-up	Total
Boscardim et al. [14]	*		*	*	*		*	*	6
Chou [15]	*		*	*	*		*	*	6
Hsu et al. [16]	*		*	*	*		*	*	6
Lin [18]	*		*	*	*		*	*	6
Lin et al. [19]	*		*	*	*		*	*	6
Munia et al. [10]	*		*	*	*		*	*	6
Raposio et al. [20]	*		*	*	*		*	*	6
Rex et al. [21]	*		*	*	*		*	*	6
Ribas Milanez de Campos et al. [22]	*		*	*	*		*	*	6
Schmidt et al. [11]	*		*	*	*		*	*	6
Sciuchetti et al. [12]	*		*	*	*		*	*	6
Leao et al. [17]	*		*	*	*			*	5
Zacherl et al. [13]	*		*	*	*		*	*	6

*Presence of the dominion evaluated.

### Results of the outcome satisfaction

**Figure 2.**
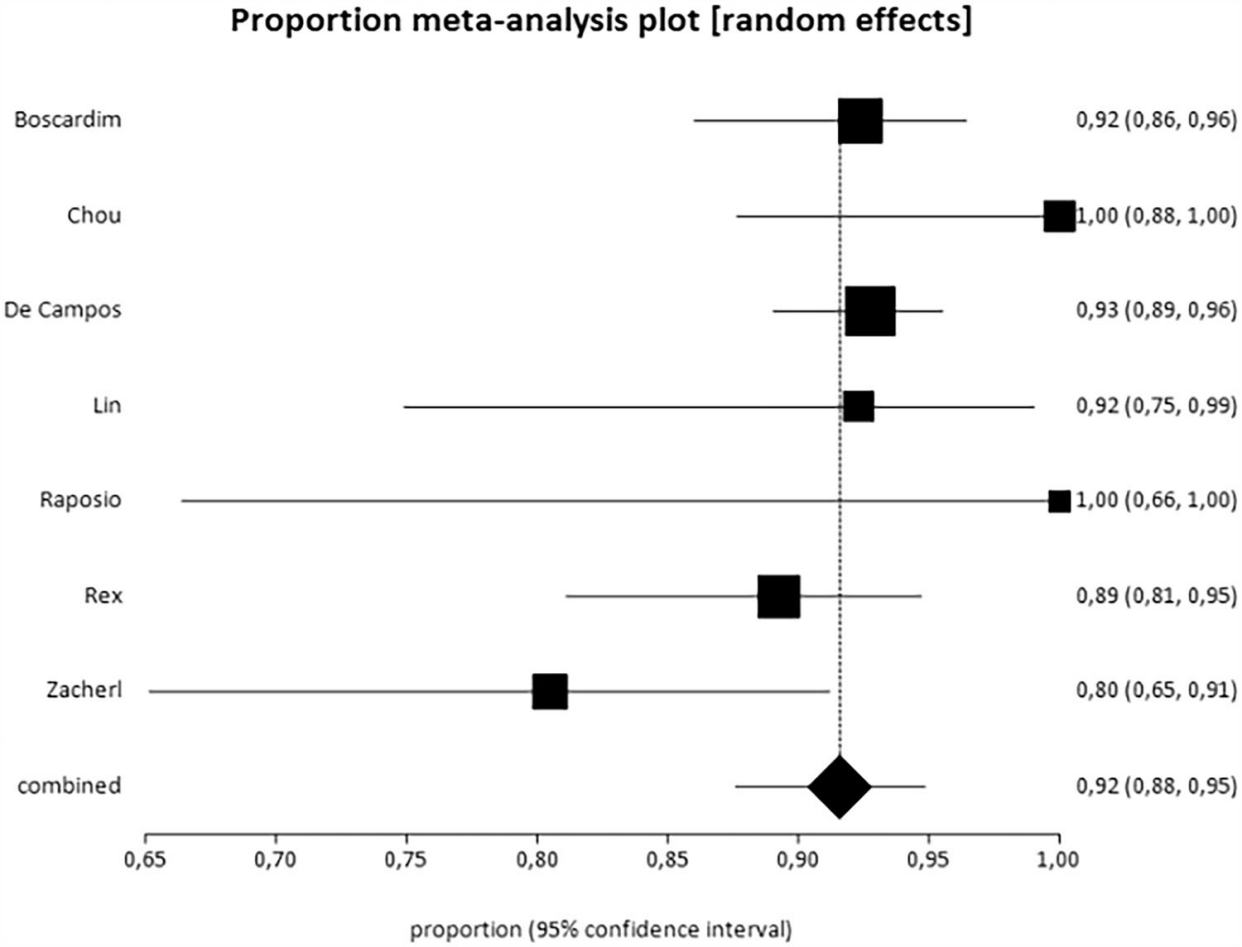
Results of the overall satisfaction outcome. The overall satisfaction rate was 92% (95% CI = 88–95%, *I*^2^=47.5%).

Satisfaction after surgical treatment was assessed in eight studies [10,13–15,18,20–22], thus totalling 655 patients, i.e. 44.77% of the total sample. All of them presented the data dichotomously or trichotomously, with the exception of the study by Munia et al. [10], in which the results were expressed as mean values; therefore, this study was not included. Figure 2 shows the results of the proportional meta-analysis. For the analysis, the results of patients satisfied and partially satisfied with the surgery were combined because surgery had a positive impact on quality of life in this group.

Boscardim et al. [14] analysed the results obtained with the surgery in the seventh postoperative period and after 12 months. At the initial assessment, 93.22% of the patients were satisfied and 6.77% were indifferent or dissatisfied with the surgery. After 12 months, 88.13% of the patients were satisfied and 11.8% were indifferent or dissatisfied with the surgery.

### Results for the outcome control of symptoms

**Figure 3.**
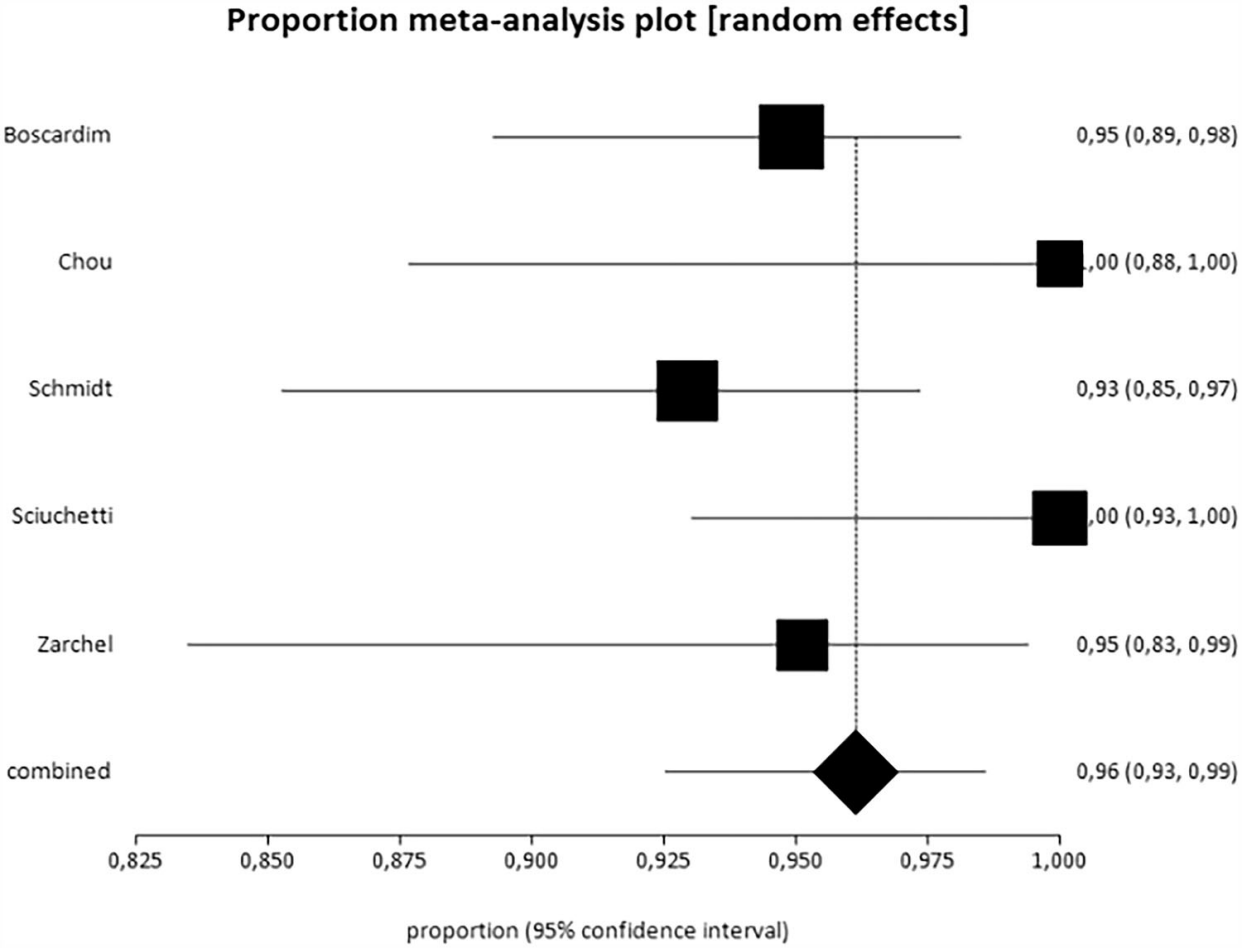
Results of the outcome total control of symptoms. The rate of symptom control was 96% (95% CI = 93–99%, *I*^2^=48.2%).

Data from six studies were used for this outcome [11–16]. The study by Leao et al. [17] was not included because it was impossible to clearly determine the exact number of patients with controlled symptoms. Thus, the data of 492 patients were analysed, which corresponds to 33.62% of the sample. Figure 3 shows the results obtained for the outcome control of symptoms. For this analysis, similar to the satisfaction outcome analysis, the results of patients with total and partial control of symptoms were combined. The study by Hsu et al. [16] significantly diverged from the others, which raised heterogeneity to 90% and prevented the combination of the studies. Therefore, this study was withdrawn from the meta-analysis, which lowered heterogeneity to 48.2%.

Boscardim et al. [14] compared early and late results during follow-up. The total control of symptoms was 83.89% at the initial assessment and 68.64% after the follow-up. The rates of partial control or no control were 16.10% in the first assessment and 31.35% after 12 months. In the study by Zacherl et al. [13] whose average follow-up time was 16.1 years, total symptom control was 85.36% in the first evaluation and 68.29% in the late evaluation, with a reduction in the long-term symptom control rate.

### Results of the outcome compensatory sweating

The presence of compensatory sweating was reported in eight articles [10,14–19,22]; however, it was impossible to analyse the data of Hsu et al. [16] because there was no quantification of compensatory sweating. Thus, data from 1013 patients (69.24%) were analysed. In 223 patients (22.01%), there was no manifestation of compensatory sweating after surgery. Among those who exhibited compensatory sweating, 390 (38.49%) reported mild symptoms, 306 (30.20%) had moderate symptoms and 94 (9.27%) had severe symptoms. No meta-analysis was performed because of the high heterogeneity among the studies (97.1%), which prevented combining the studies.

### Results of the outcome complications

The occurrence of complications was reported in seven studies [10–12,17–19,22]. As the studies of Schmidt et al. [11] and Sciuchetti et al. [12] included patients with hyperhidrosis in areas other than the axilla, they were not included in the analysis. Leao et al. [17] and Munia et al. [10] did not observe any complications in the evaluated group. Ribas Milanez de Campos et al. [22] evaluated 276 patients and reported the presence of residual pneumothorax in eight patients, five cases of atelectasis, one case of pain at the trochanter insertion site, one case of hemothorax due to venous bleeding intercostal and one case transient bradycardia. In the study by Lin [18], one of the 26 analysed patients had atelectasis and another experienced recurrence of symptoms during follow-up. Lin et al. [19] analysed 480 patients and reported 80 cases of recurrence during follow-up, which ranged from one to five years. There were no serious complications or deaths in any study.

### Subgroup analysis

The studies were divided into two subgroups, with the first including the studies in which the sympathetic trunk resection was performed at higher levels, and the second encompassing studies in which it was performed at lower levels. The analysis was performed for the outcomes satisfaction, symptom control and compensatory sweating.

#### Outcome satisfaction

All studies present in the general analysis were included with the exception of the study by Munia et al. [10] in which the results were expressed as means. Table 3 summarizes the results.

**Table 3.** Analysis of the outcome satisfaction according to the level of sympathectomy.

Resection level							
High				Low			
Author	Level	*N*		Author	Level	*N*	
		S	I			S	I
Lin	T3/T4	24	2	Boscardim	T4/T5	109	9
Rex	T2/T4	83	10	Chou	T5	28	0
Ribas Milanez de Campos	T3/T4_T3	256	20	Fox	T4	9	0
Zacherl	T1/T4	33	8				
Total		396	40			146	9

*N*: total number of patients; S: satisfied patients; I: dissatisfied patients.

**Figure 4.**
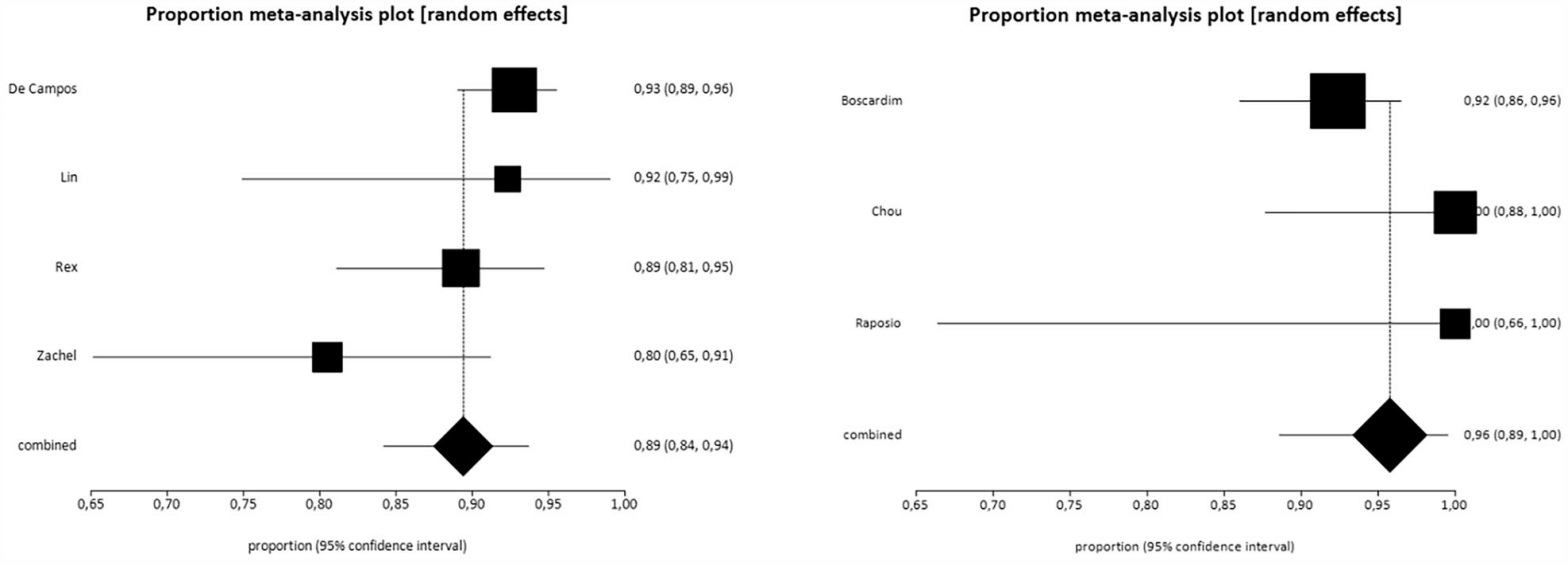
Forest plot of the outcome satisfaction in high resection levels (A) satisfaction rate 89% (95% CI = 84–95%, *I*^2^=47.7%) and low (B) satisfaction rate 96% (95% CI = 89–100%, *I*^2^=45.9%).

**Figure 5.**
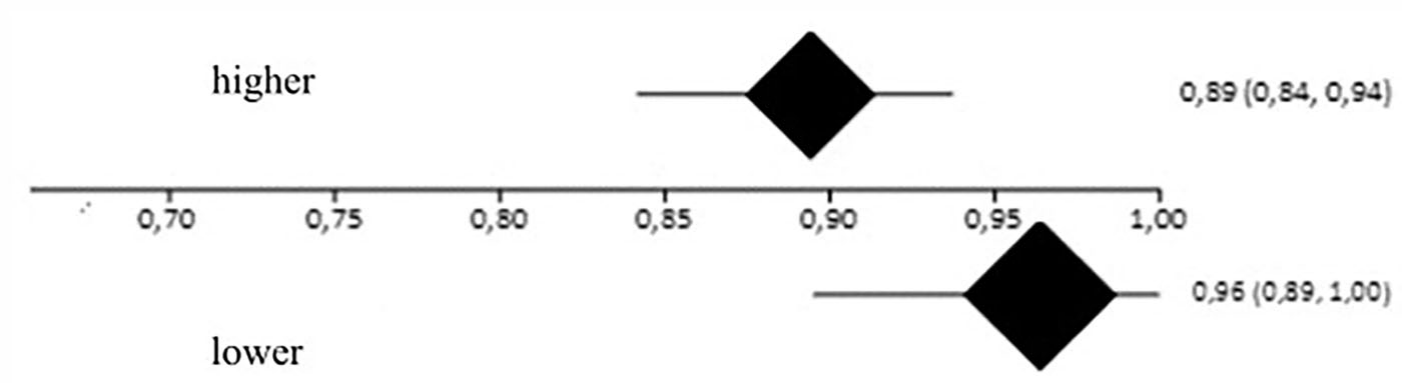
Interpretation of meta-analysis of the outcome satisfaction. The overlap of the confidence intervals indicates that there were no differences between the groups.

The satisfaction rate was 89% (95% CI = 84–94%, *I*^2^=47.7%) and 96% (95% CI = 89–100%, *I*^2^=45.9%) among patients with higher and lower resection levels, respectively (Figure 4). As there was an overlap between the confidence intervals, there were no differences between the high and low resection levels (Figure 5).

#### Outcome control of symptoms

The evaluation of the symptom control outcome was performed in five studies [11–15]. Once again, the study by Hsu et al. [16] was removed from the meta-analysis because of high heterogeneity. Table 4 summarizes the results of the studies.

**Table 4.** Analysis of the outcome control of symptoms according to the level of sympathectomy.

Resection level									
High					Low				
Author	Level	*N*			Author	Level	*N*		
		P	A				P	A	
Schmidt	T2/T4_T3/T5	79		4	Boscardim	T4/T5	112		6
Sciuchetti	T3/T4	51		0	Chou	T5	28		0
Zacherl	T1/T4	39		2					
Total		169		6			140		6

*N*: total number of patients; P: patients with controlled symptoms; A: patients with uncontrolled symptoms.

**Figure 6.**
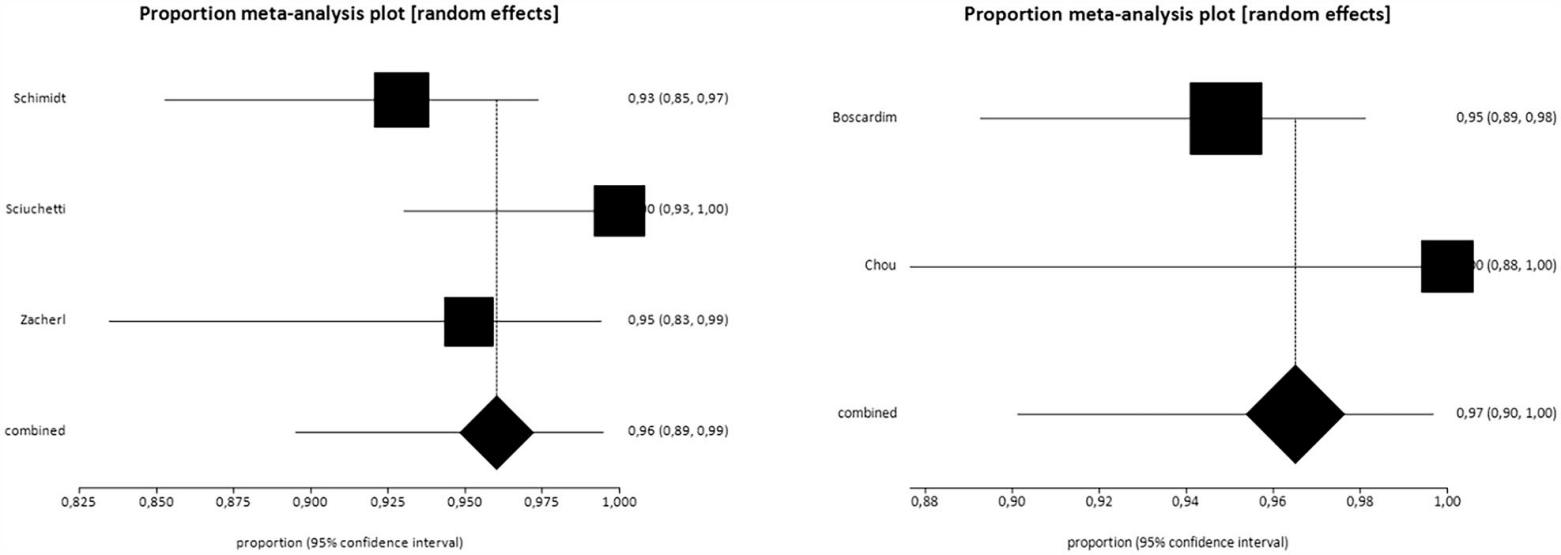
Forest plot of the outcome control of symptoms in high resections (A) control rate 96% (95% CI = 89–99%, *I*^2^=65.9%) and low (B) 97% control rate (95% CI = 90–100%, *I*^2^=46.3%).

**Figure 7.**
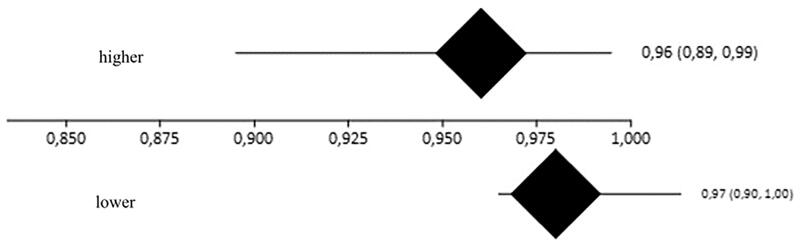
Interpretation of meta-analysis of the outcome control of symptoms. The overlap between confidence intervals shows that there were no differences between the groups.

The rates of total symptom control were 96% (95% CI = 89–99%, *I*^2^=65.9%) and 97% (95% CI = 90–100%, *I*^2^=46.3%) in patients who underwent sympathectomy at higher and lower levels, respectively (Figure 6). The overlap between confidence intervals indicates that there was no difference between groups (Figure 7).

#### Outcome compensatory sweating

The analysis of the outcome compensatory sweating included all studies, with the exception of the publication of Hsu et al. [16] who did not report the effect of compensatory sweating on patients' quality of life. For the evaluation, the absent and mild results were combined and compared to the moderate and severe results considering the impact on quality of life. Table 5 summarizes the results of the studies.

**Table 5.** Analysis of the outcome compensatory sweating as per the level of sympathectomy.

Resection level							
High				Low			
Author	Level	*N*		Author	Level	*N*	
		A	P			A	P
Lin	T3/T4	25	1	Boscardim	T4/T5	96	22
Lin	T3/T4	288	192	Chou	T5	28	0
Munia	T3/T4	2	29	Munia	T4	19	14
Ribas Milanez de Campos	T3/T4_T3	137	139				
Leao	T2/T4	18	3				
Total		470	364			143	36

*N*: total number of patients; A: patients without compensatory sweating; P: patients with compensatory sweating.

Similar to the analysis of the overall outcome, heterogeneity among the studies in this analysis was >90% for the two groups, which made it impossible to compare the results; therefore, proportional meta-analysis was not performed.

### Assessment of the quality of evidence

The quality of evidence evaluated using the GRADE system is presented in Table 6.

**Table 6.** Quality of evidence for the outcomes satisfaction and control of symptoms in video-assisted thoracoscopic sympathectomy for treating pure axillary hyperhidrosis.

Outcome	Study design	Risk of bias	Inconsistency	Indirect evidence	Imprecision	Events	No of participants (studies)	Proportion effect (95% CI)	Quality of evidence
Satisfaction	Observational	Serious^a^	Very serious^b^	Low	Not serious^c^	542	591 (7)	0.92 (0.88–0.95)	⨁◯◯◯ very low
Symptom control	Observational	Serious^a^	Very serious^b^	Low	Not serious^c^	309	323 (5)	0.96 (0.93–0.99)	⨁◯◯◯ very low

CI: confidence interval.

Very low quality: any estimate of the effect of the intervention is very uncertain.

^a^ Risk inherent to the nature of the included studies, subject to biases of interviewer, memory, performance and confusion.

^b^ High risk of inconsistencies due to high levels of heterogeneity.

^c^ The confidence intervals were relatively narrow.

## Discussion

Axillary hyperhidrosis is a disorder that, although benign, has considerable impact on the quality of life of the affected individuals [6]. Treatment is usually performed in a staged manner, and a surgical approach is indicated after failure of topical and less invasive treatments, as local surgery. [8]. To date, there has been no consensus on the optimal surgical therapeutic option for patients with pure axillary hyperhidrosis [4]. In the literature, the success rates of sympathectomy for treating primary facial, palmar and axillary hyperhidrosis vary between 68% and 100% and immediate satisfaction rates can reach 100% [7,8]. In this review, the rates of satisfaction and control of symptoms after follow-up were 90%. A study not included in the meta-analysis [16] demonstrated values below the average in the literature, with a rate of symptom control of <50%. Although certain studies show a decrease of up to 65% in satisfaction rates over time [8], some researchers did not identify a significant reduction after follow-up. In the study by de Campos et al. [6], the data of 513 patients after 10 years of follow-up did not show a significant reduction relative to the satisfaction results obtained immediately after surgery. Similarly, Wolosker et al. [23] did not observe a reduction in satisfaction rates after a period of five years. In this review, all studies comparing early and late results showed a decrease in the rates of satisfaction and symptom control [13,14]. The results of axillary resection surgeries (axillary tissue excision, suction and curettage) are similar. Zhao et al. [24] analysed the results of 396 patients who underwent surgical resection of axillary tissue and obtained a patient satisfaction of 87.1% after five years of follow-up. In the study by Bechara et al. [25], the satisfaction rate nine months after axillary curettage was 78.4%. The primary complication of thoracic sympathectomy that may compromise quality of life after surgery is compensatory sweating, which can reach 80% of palmar cases and up to 100% of cases of axillary hyperhidrosis [8]. As reported by de Campos et al. [6], compensatory sweating is the primary cause of dissatisfaction after surgery. Although it does not tend to regress over time, it only has a negative impact quality of life if it compromises daily activities. However, compensatory sweating should be a perennial concern and all patients should be advised about the possibility of its occurrence [6,8]. Local axillary surgery rarely leads to compensatory sweating [7] but other complications are relatively common. Bechara et al. [25] did not report any serious complications but patients developed haematomas (76.5%), skin erosion (27.5%), bridles (21.6%), seromas (13.7%), dysesthesia (11.8%) and hair loss (7.8%). In the study by Zhao et al. [24], 11.4% of patients had important side effects after follow-up, the primary ones being scars, keloids and recurrence of bromhidrosis. The high heterogeneity among the reviewed studies that evaluated the presence of compensatory sweating did not allow a meta-analysis. Note that 78% of patients had compensatory sweating and in 50% of these symptoms negatively impacted quality of life. These data contrast with those presented by de Campos et al. [6] whose result of compensatory sweating after sympathectomy for treating primary hyperhidrosis was 94.5%. However, in this group, only 1.7% of patients reported dissatisfaction with surgery, but in the sample used in this study only 11.6% of patients had pure axillary hyperhidrosis.

A factor that has an impact on the results of thoracic sympathectomy is the level at which the sympathetic trunk section is made. Sections made at lower levels show good results with less compensatory effect [6,8]. A meta-analysis including prospective and randomized studies that evaluated the effect of sympathetic resection height in patients with palmar and axillary symptoms demonstrated a higher risk of compensatory sweating in patients undergoing resection at higher levels (RR 7.25, 95% CI 2.30–22.84, *I*^2^=0) [26]. In this review, the subgroup analysis demonstrated no differences in satisfaction rates, symptom control and compensatory sweating between the groups that underwent high and low resections of the sympathetic trunk. These discrepancies between the reviews are probably attributed to differences between the samples and the type of primary study used in the analyses.

### Limitations of the study

Only four studies in this review were prospective and only one study used consecutive allocation, a procedure that predisposes studies to selection bias. Note that not all publications made it clear what the selection criteria were; however, it was always evident that patients should have pure axillary hyperhidrosis. Nevertheless, this fact does not prevent the generalization of the results because the study population represents a very specific group. Another limitation is the fact that postoperative evaluation was not performed with the same instrument in all studies. Moreover, several studies are retrospective, which can create a memory bias at the time the patients answered the researchers’ questions. Note that loss of patients to follow-up in the studies was negligible and the flow of patients was well described, which minimizes the chances of selection bias. To summarize, the quality of evidence for all outcomes was very low because of the lack of prospective and randomized studies.

## Conclusions

This review shows that surgical treatment of pure axillary hyperhidrosis can be safely performed through thoracic sympathectomy, with good long-term results and low rates of complications, but the estimation of this effect is very uncertain because the quality of evidence is very low. Moreover, proportional meta-analysis demonstrated no differences between high and low resection levels for all analysed outcomes.

## Data Availability

The data that support the findings of this study, such as the list of articles found, the articles data, the list of excluded articles and any other are available from the corresponding author, Felisberto Jr G, upon request.
